# Comparison of Islet Function in People With Different Glucose Metabolic States

**DOI:** 10.2174/0118715303436762260508092256

**Published:** 2026-05-21

**Authors:** Guiling Li, Yixian Liu, Xiaoxuan Liu, Huijing Luo, Huimin Zhou, Xu Huang, Qian He, Jinhong Li, Yanv Ren, Xiaofang Zhang, Zuoliang Dong

**Affiliations:** 1 Department of Clinical Laboratory, Tianjin Medical University General Hospital, No.154 Anshan Road, Heping District, Tianjin 300052, China;; 2 Bacteriological Disease Prevention and Disinfection Institute, Hebei Province Center for Disease Prevention and Control, No. 97 Huai’an Donglu, Shijiazhuang 050021, Hebei, China;; 3 School of Medical Imaging, Tianjin Medical University, No.1 Guangdong Road, Hexi District, Tianjin, 300204, China

**Keywords:** β-cell dysfunction, impaired glucose regulation, insulin resistance, oral glucose tolerance test, type 2 diabetes mellitus, insulinogenic index

## Abstract

**Introduction:**

Type 2 Diabetes Mellitus (T2DM) is closely associated with impaired islet β-cell function. Multiple indicators can be used to evaluate islet function and insulin resistance. The aim of this study is to establish a β-cell function assessment model based on oral glucose tolerance test (OGTT) indices, providing a reference for the early diagnosis and treatment of diabetes.

**Methods:**

In this study, 30 Normal Glucose Tolerance (NGT), 43 Impaired Glucose Regulation (IGR), and 42 T2DM subjects were enrolled. The levels of blood glucose, insulin, c-peptide, and proinsulin were measured during OGTT. Insulinogenic index (IGI), C-Peptide generation Index (CPI), and proinsulinogenic index (PNI) were calculated. Fasting-state ratios, including c-peptide to glucose (CP/I), proinsulin to insulin (PI/I), and proinsulin to c-peptide (PI/CP), were calculated. The area under the curve (AUC) was analyzed for these indices and their corresponding ratios. ROC curve analysis was performed to evaluate the diagnostic value of these indicators for glucose metabolism disorders and β-cell function.

**Results:**

In the fasting state, CP/I gradually increased, while IGI and PNI gradually decreased from the NGT group to the IGR and T2DM groups. For diagnosing IGR, the AUCs of CP/I, IGI, and PNI were 0.8101, 0.762, and 0.7605; for T2DM, they were 0.8746, 0.9651, and 0.8451; and for distinguishing IGR from T2DM, they were 0.7054, 0.8200, and 0.6833, respectively.

**Discussion:**

Different indicators have different diagnostic capabilities for IGR and T2DM. The comparison of various indicators reveals pancreatic islet function across different glucose-metabolizing populations.

**Conclusion:**

CP/I, IGI, and PNI were more sensitive in reflecting impaired glucose tolerance. CP/I can reflect insulin clearance capacity, and IGI can reflect β-cell dysfunction.

## 1 INTRODUCTION

Type 2 Diabetes Mellitus (T2DM) is characterized by insulin intolerance and elevated blood glucose [1]. Insulin is secreted by β-cells and is crucial for maintaining a balance between glucose, energy, fat, and proteins. In healthy individuals, blood glucose is maintained at normal levels by a compensatory increase in insulin secretion. However, in those susceptible to T2DM, β-cell compensation diminishes, leading to glucose intolerance and, ultimately, diabetes [2, 3]. Adequately assessing β-cell function and insulin resistance status can aid in selecting treatment regimens and predicting the prognosis of patients with T2DM. Multiple models for measuring insulin resistance and β-cell function have been developed, based on dynamic and static insulin tolerance assays [4]. Each method for assessing β-cell function has its own strengths and weaknesses. Clinically, the sensitivity and specificity of each method must be considered in order to maximize efficacy [5]. Glucose-stimulation tests, including the hyperglycemic clamp test and the intravenous glucose tolerance test (IVGTT), are preferred for evaluating patients with diabetes who exhibit normal glucose tolerance [6]. The Oral Glucose Tolerance Test (OGTT) is a commonly used method for diagnosing glucose metabolism disorders and estimating β-cell function, and it provides a more accurate reflection of physiological changes in glucose and insulin than other glucose-stimulation tests [4].

Proinsulin (PI) is a precursor of insulin that undergoes various intracellular processes, including oxidative folding, intracellular transport, and enzymatic processing, to be converted into insulin. An abnormal elevation in the circulating PI and ratio of PI to insulin (PI/I) are commonly used indicators of pancreatic β-cell dysfunction and insulin resistance in patients with prediabetes and T2DM [6]. Insulin and c-peptide are both secreted in equal amounts by the pancreas; however, the liver absorbs approximately half of the secreted insulin, whereas the tissues barely absorb c-peptide and it remains in circulation. Therefore, measuring circulating c-peptide levels provides a more precise assessment of insulin secretory capacity [7]. In addition to proinsulin and c-peptide, there are a variety of calculations that can be made to determine pancreatic β-cell function. The homeostatic model assesses insulin resistance and β-cell function using fasting insulin and blood glucose levels. It is a correction to the fasting insulin/fasting plasma glucose simplification index [4, 5] and is simple and easy to use; however, it is difficult to reflect the true relationship between insulin and glucose using only a single point [5]. The insulinogenic index (IGI) quantifies the β-cell response based on changes in plasma glucose levels [4]. The Disposition Index (DI) is the smallest model for assessing β-cell function and is calculated from the product of insulin sensitivity and insulin secretion. When β-cell function fails, the DI will decrease [4, 8]. There are many other methods of assessing β-cell function [9-11].

In this study, glucose, insulin, c-peptide, and proinsulin levels were examined in both fasting and post-glycemic loading states. The aim of the study is to develop models to assess pancreatic β-cell function and insulin resistance using the levels of four indices during the OGTT. These models were evaluated using data from individuals with Normal Glucose Tolerance (NGT), Impaired Glucose Regulation (IGR), and T2DM. Simultaneously, the capacity of these indicators to assess pancreatic β-cell function and insulin resistance was explored to provide an evidence base for clinical diagnosis and treatment.

## 2 MATERIALS AND METHODS

This study is a cross-sectional study.

### 2.1 Study Population

Individuals who received an OGTT examination for the first time in Tianjin Medical University General Hospital between March and August 2022 were enrolled in this study. Patients were divided into NGT, IGR, and T2DM groups. The criteria for inclusion in the T2DM and IGR groups were as follows: (1) patients who had been initially diagnosed with T2DM, Impaired Fasting Glucose (IFG), or Impaired Glucose Tolerance (IGT); (2) those aged ≥18 years; and (3) those who met the diagnostic criteria as shown in Table **1** [12].

The control (NGT) group was required to meet the following criteria: (1) absence of specific diseases and malignancies, renal failure, heart disease, thyroid disease, and other inflammatory diseases; (2) diagnostic criteria as shown in Table **1**; and (3) no significant abnormalities in blood count, urine routine, and biochemical tests. The exclusion criteria for all individuals were as follows: (1) previous diagnosis of diabetes mellitus, acute or chronic infectious diseases, or history of severe liver or kidney disease; or (2) pregnancy or lactation. The final sample consisted of 115 participants: 30 with NGT, 43 with IGR (including individuals with IFG and IGT), and 42 with T2DM. List the number of people of each gender according to the SAGER checklist Table **2**. All patients were informed of this study and provided their consent. Ethical approval for this study was obtained from the ethical committee of Tianjin Medical University General Hospital. Ethics committee approval number is IRB2023-WZ-187.

### 2.2 Blood Sample Collection and Storage

Participants fasted overnight (at least 8 hours) before undergoing the OGTT, as required by the Chinese Guidelines for the Prevention and Treatment of Type 2 Diabetes (2020 Edition). The OGTT involved the following procedure: (1) 75 g of anhydrous dextrose powder was dissolved in 300 mL of water and administered orally to participants within 5 min, starting between 7:00 and 9:00 a.m. (2) Participants refrained from tea or coffee consumption, smoking, or strenuous exercise during the test period. (3) Daily carbohydrate intake was at least 150 g for three days prior to the test. (4) Medications that may affect the OGTT, such as birth control pills, diuretics, or phenytoin sodium, were discontinued 3-7 days prior to the test. (5) Blood was collected in sample tubes in the fasted state and at 30, 60, 120, and 180 min after glucose loading. Subsequently, the samples were centrifuged at 1, 200 g for 10 min, and the supernatant was collected. Glucose was detected using a fully automated biochemistry analyzer (LST008AS) and the accompanying kit (Hitachi, Japan). Insulin was detected using an ARCHITECT i2000SR chemiluminescence analyzer and the accompanying kit (Abbott Laboratories, Abbott Park, USA). C-peptide and proinsulin were detected using a fully automated chemiluminescence immunoassay analyzer, Maglumi 2000 Plus, and the accompanying kit (Shenzhen New Industry Company, China). Internationally agreed calibrators (WHO 84/150) were used for c-peptide. The results of the c-peptide assay in our study were rigorously traced to ensure a certain degree of stability.

### 2.3 Insulin Resistance and β-cell Function Calculation

Insulin resistance and insulin clearance were assessed using the homeostatic model assessment for insulin resistance (HOMA-IR) and the c-peptide-to-insulin ratio (CP/I) during fasting [8].

β-cell function model: The homeostasis model assessment-β (HOMA-β) was calculated using fasting insulin and glucose levels. The IGI (∆I30/∆G30) was calculated using 0 min and 30 min samples. Modeled after IGI using 0 min and 30 min samples to calculate c-peptide generation index (CPI: ∆C30/∆G30) and proinsulinogenic index (PNI: ∆PI30/∆G30). The area under the curve (AUC) for glucose, insulin, c-peptide, and proinsulin (G-AUC, I-AUC, CP-AUC, and PI-AUC) was calculated using the trapezoidal rule over the entire 180 min sampling period. The ratios of proinsulin to insulin and c-peptide (PI/I and PI/CP, respectively) were calculated in the fasting state. The ratios of the proinsulin AUC to the insulin and c-peptide AUCs (PI-AUC/I-AUC and PI-AUC/CP-AUC, respectively) were calculated. The PI-AUC/G-AUC, CP-AUC/G-AUC, and I-AUC/G-AUC ratios were calculated.

### 2.4 Data Analysis

All analyses were conducted using IBM SPSS 26.0 and GraphPad Prism 9.3.0. Nonparametric tests were used to compare any two groups, with statistical significance set at *P* < 0.05. The diagnostic efficacy of the sensitivity index for glucose tolerance in identifying dysglycemia was assessed using the receiver operating characteristic (ROC) curve and its corresponding AUC.

## 3 RESULTS

### 3.1 Basic Study Population Information

Basic information about the study individuals is shown in Table **2**. Compared with the NGT group, the proportion of males was higher in both the T2DM and IGR groups. The Body Mass Index (BMI) of the IGR group was higher than that of the NGT and T2DM groups. Furthermore, insulin levels in the IGR group were higher than those in the other groups at 120 min, whereas there were no significant differences in fasting insulin levels among the groups. A comparison of blood glucose, insulin, C-peptide, and proinsulin levels at different time points during the OGTT across groups is shown in (Supplementary Figure **1**)). The C-peptide levels in patients with IGR or T2DM were higher than those in individuals with NGT (Supplementary Figure **1c**). However, between 30 and 180 min, there were no significant differences in c-peptide levels between the IGR and T2DM groups. Insulin and proinsulin levels were not significantly different among the three groups.

### 3.2 Oral Glucose Tolerance Post-curve Study

**Fig. (1):**
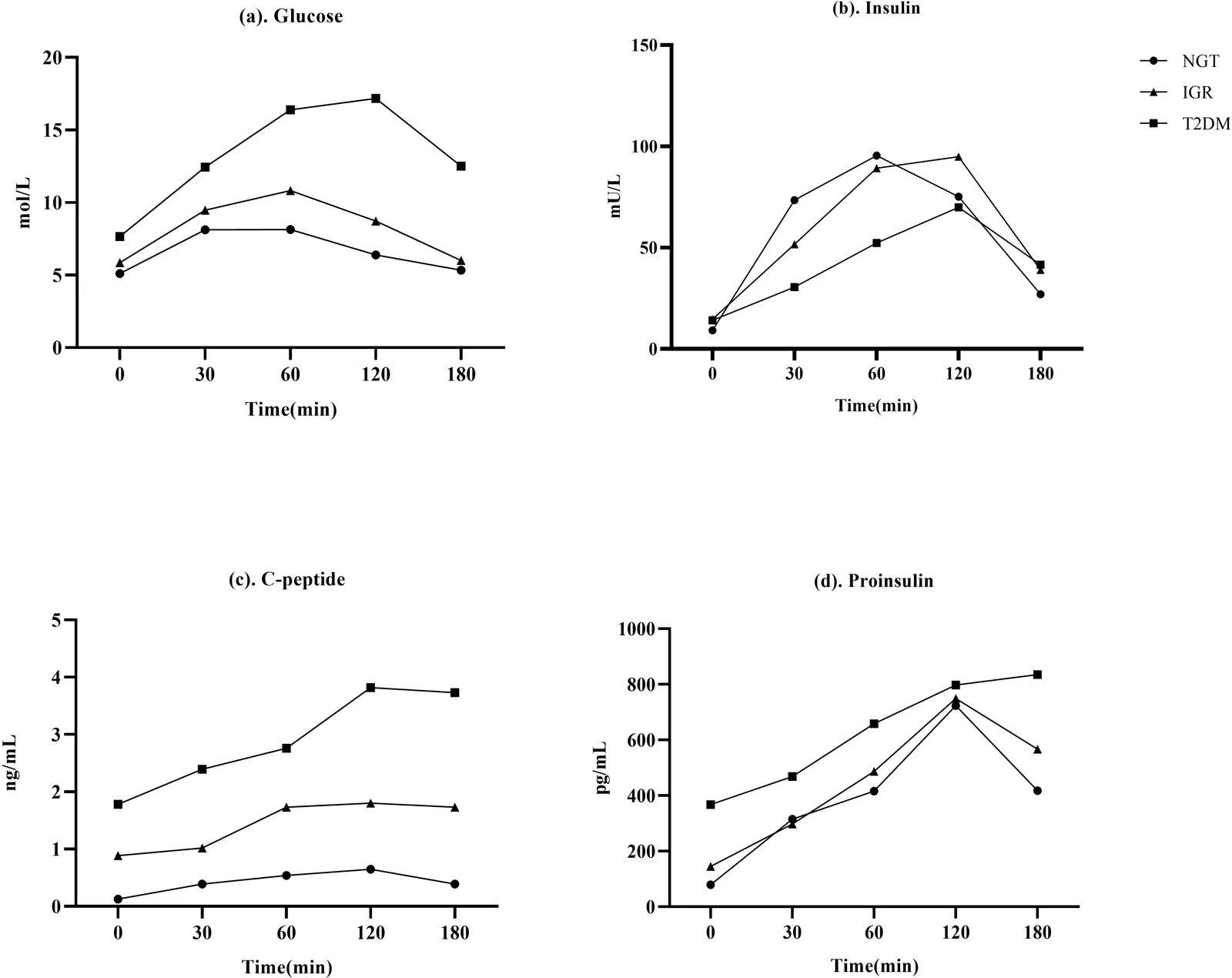
Changes in glucose, insulin, c-peptide, and proinsulin profiles during the OGTT among groups. (**a**) Glucose; (**b**) Insulin (**c**) C-peptide; and (**d**) Proinsulin. The dots in the graphs indicate the median for each indicator. NGT: normal glucose tolerance, IGR: impaired glucose regulation, T2DM: type 2 diabetes mellitus.

In the NGT group, glucose peaked at 60 minutes (8.135 mmol/L) before returning to normal at 120 min (Fig. **1a**). Insulin was the highest at 60 min (95.400 mU/L), with a peak value approximately 10 times that of fasting insulin (Fig. **1b**). Both c-peptide and proinsulin peaked at 120 minutes (0.646 ng/mL and 677.500 pg/mL, respectively) (Fig. **1c** and **d**). In the IGR group, glucose peaked at 60 min (10.820 mmol/L) and returned to normal levels at 180 min (Fig. **1a**). Insulin reached its peak level later than that in the NGT group, reaching its highest point at 120 min (95.000 mU/L) (Fig. **1b**). C-peptide and proinsulin peaked at 120 min (1.800 ng/mL, 774.000 pg/mL) (Fig. **1c** and **d**). In the T2DM group, insulin levels peaked at 120 min (66.900 mU/L), and glucose, c-peptide, and proinsulin levels consistently exceeded those in the other groups. The trends of c-peptide and proinsulin levels remained consistent across all groups (Fig. **1**). The AUCs for glucose, insulin, C-peptide, and proinsulin during the OGTT were compared across the groups (Supplementary Figure **2**)). The AUC of glucose levels in patients with T2DM was higher than in the IGR and NGT groups, and the AUC in the IGR group was higher than in the NGT group. The IGR and T2DM groups displayed higher AUC values for c-peptide than in the NGT group. There were no significant differences in the AUC for proinsulin across the different groups.

### 3.3 Modeling of Insulin Resistance and Pancreatic Β- Cell Function

The modeling of insulin resistance and β-cell function in each group is shown in Table **3**. There were no differences in the HOMA-IR and HOMA-β indices between the NGT and IGR groups. The CP/I ratio increased as glucose regulatory status declined. Additionally, the IGI and PNI decreased with disease progression across the NGT, IGR, and T2DM groups. The P/I ratio was higher in the T2DM group than in the NGT and IGR groups. Fasting PI/CP decreased significantly in the IGR and T2DM groups compared to the NGT group. The PI-AUC/I-AUC was higher in the T2DM group than in the other groups. The PI-AUC/CP-AUC was lower in the IGR and T2DM groups than in the NGT group, whereas the I-AUC/G-AUC was lower in the T2DM group than in the other groups. The CP-AUC/G-AUC increased in the IGR group. CP-AUC/G-AUC increased in the IGR group, and PI-AUC/G-AUC decreased in the T2DM group compared to the NGT group.

### 3.4 Diagnostic Power of CP/I, IGI, and PNI for Disorders of Glucose Metabolism

**Fig. (2):**
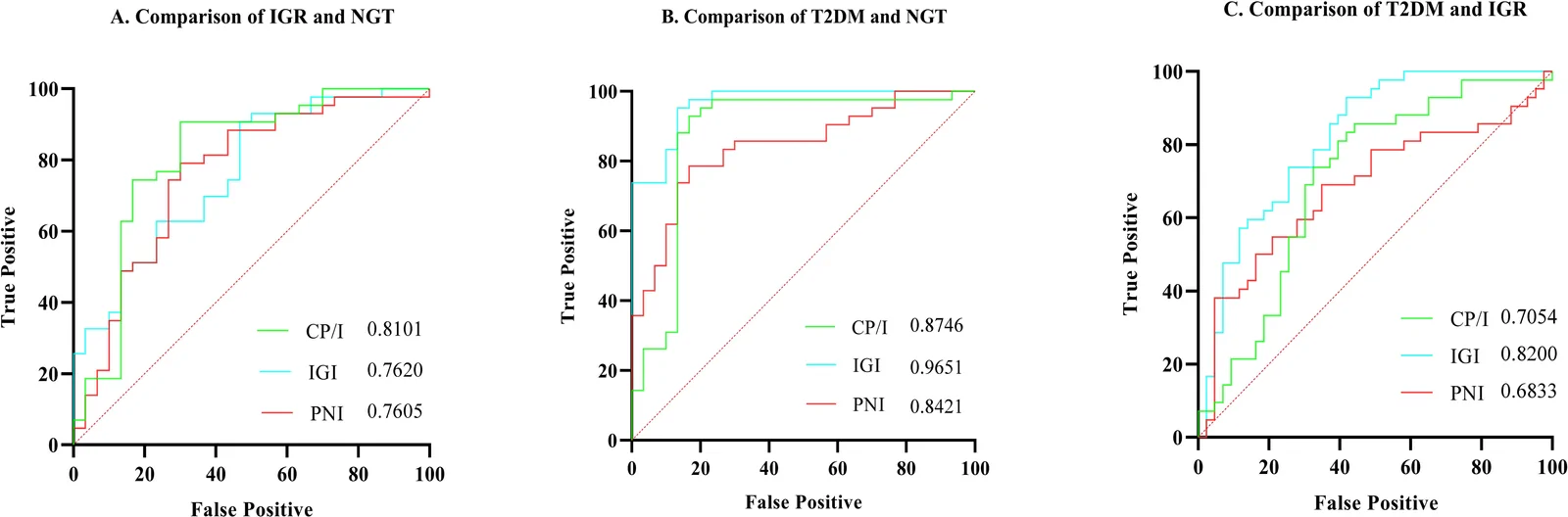
Predictive effect of CP/I, IGI, and PNI on diabetes. (**a**) Efficacy of CP/I, IGI, and PNI relative to NGT in diagnosing impaired IGR. (**b**) Efficacy of CP/I, IGI and PNI relative to NGT in diagnosing T2DM. (**c**) Efficacy of CP/I, IGI, and PNI relative to IGR in diagnosing T2DM. NGT: Normal glucose tolerance, IGR: impaired glucose regulation, T2DM: type 2 diabetes mellitus.

In order to determine these metrics for evaluating islet function and clearance, the CP/I, IGI, and PNI displayed significant differences among the NGT, IGR, and T2DM groups. In this study, the diagnostic efficacy of these three metrics for evaluating pancreatic β-cell function and insulin clearance was assessed based on their performance in diagnosing glucose metabolism disorders. These three indicators were analyzed using ROC curves and their corresponding AUC values (Fig. **2**). For the diagnosis of IGR, the AUCs for CP/I, IGI, and PNI were 0.8101, 0.762, and 0.7605, respectively (Fig. **2a**). However, for the diagnosis of T2DM, the AUCs for CP/I, IGI, and PNI were 0.8746, 0.9651, and 0.8451, respectively (Fig. **2b**). When distinguishing between IGR and T2DM, the AUCs for CP/I, IGI, and PNI were 0.7054, 0.8200, and 0.6833, respectively (Fig. **2c**).

## 4 DISCUSSION

Insulin is the core hormone that regulates glucose, lipid, and protein metabolism in the body [12]. Following the ingestion of an oral glucose load or a mixed meal, the plasma glucose concentration increases, which in turn stimulates pancreatic β-cells to secrete insulin. At this point, the resulting hyperinsulinemia acts in synergy with hyperglycemia to suppress endogenous glucose production and promote glucose uptake by muscle, liver, and adipocytes, thereby enhancing glucose uptake in muscle tissue [13]. The pathogenesis of T2DM involves dual defects: insulin resistance and β-cell dysfunction [14]. It leads to insufficient insulin secretion, impaired cellular response to extracellular insulin, and/or compromised glucose metabolism, resulting in chronic elevation of blood glucose levels [15]. Early assessment of insulin resistance and β-cell function is particularly important for diagnosing and treating T2DM. In addition to the traditional indicators of insulin and glucose, growing attention is being paid to novel biomarkers derived from the OGTT that can reflect pancreatic β-cell function [16]. In this study, glucose, insulin, c-peptide, and proinsulin levels were measured at different time points during the OGTT across different groups. Different models for assessing insulin resistance and pancreatic β-cell function were calculated based on these indicators, and their ability to diagnose glucose metabolism disorders was analyzed.

The present study found that the proportion of males in both the T2DM and IGR groups was higher than in the NGT group, consistent with previous studies showing that the prevalence of T2DM was higher in males than in females [17]. The subjects' BMI increases in the prediabetes stage and decreases after a confirmed diagnosis of diabetes. Comparison of blood glucose, insulin, c-peptide, and proinsulin levels at different time points revealed a noteworthy difference in fasting c-peptide levels among the NGT, IGR, and T2DM groups. C-peptide is released 1:1 with insulin and is resistant to liver uptake [18]. Consequently, c-peptide levels have been used to estimate insulin production and storage in clinical settings [19]. Hepatic insulin clearance is impaired in patients with impaired glucose tolerance [20]. Therefore, changes in insulin do not coincide with changes in c-peptide and proinsulin. A universal elevation of C-peptide and proinsulin curves was observed in the T2DM group during the OGTT, relative to the other groups. Previous studies have demonstrated a full-segment elevation of the proinsulin curve in OGTT, indicating a high risk of T2DM [21]. At high blood glucose levels, β-cells accelerate insulin secretion, leading to the release of unprocessed proinsulin into the bloodstream [2], which results in elevated serum proinsulin levels in individuals with T2DM. After processing, proinsulin is cleaved into insulin and C-peptide. Because c-peptide is minimally taken up after its release, the trends of proinsulin and c-peptide during the OGTT closely parallel each other, with both peaking at 120 min. However, in the present study, no significant difference in proinsulin levels was observed between the IGR and NGT groups during the OGTT, which is inconsistent with prior research [22].

HOMA-IR and HOMA-β are commonly used static models for assessing insulin resistance and pancreatic β-cell function. However, in our study, they were unable to differentiate between the NGT and IGR groups. This may be due to HOMA-β underestimating the severity of β-cell defects in cases of impaired glucose tolerance [23]. CP/I measures the ability of the liver to absorb insulin and assesses insulin clearance [24]. Therefore, the gradual increase in CP/I from the NGT and IGR groups to the T2DM group suggests increased hepatic insulin uptake [25]. In response to hyperglycemia, β-cells increase insulin secretion, and insulin-clearing tissues become less efficient at clearing insulin; this mechanism is disrupted in patients with T2DM [26].

Fasting PI/I and PI/CP can reflect β-cell mitochondrial pressure, but they do not reflect glucose intolerance well and have limitations [27, 28]. Calculating changes in insulin, c-peptide, and proinsulin levels 30 minutes after glucose stimulation allows quantification of β-cell responses based on changes in circulating glucose levels [4], providing a more accurate representation of glucose intolerance. For the first time, the proinsulin index—derived from the change in proinsulin levels at 30 minutes after oral glucose administration—was calculated on the basis of IGI. In this study, the gradual decrease in IGI and PNI from the NGT and IGR groups to the T2DM group suggests insufficient insulin and proinsulin secretion, that is, dysfunctional β-cells. G-AUC, I-AUC, CP-AUC, and PI-AUC reflected the overall levels of glucose, insulin, c-peptide, and proinsulin, respectively, over the 3 h duration of the OGTT. Although higher proinsulin levels were observed in the T2DM group relative to the other groups throughout the entire course of the OGTT, no significant differences were detected in the AUC of proinsulin levels across all groups. This led to no significant difference (P > 0.05) in the overall proinsulin levels between the IGR and T2DM groups and the NGT group during the OGTT.

Few studies have previously assessed pancreatic β-cell function using ratios of the proinsulin AUC to the insulin and c-peptide AUCs. Although this study calculated these two metrics, the results were unsatisfactory in effectively differentiating between populations with varying islet β-cell functional states. The ratio of insulin and glucose AUCs during the OGTT has been used to evaluate insulin secretory function [29]. Hepatic uptake of insulin compromises the capacity of this metric to accurately reflect pancreatic β-cell secretory function; therefore, the ratios of the AUCs of C-peptide and proinsulin to the AUC of glucose were calculated simultaneously in the present study. However, none of these metrics effectively reflected glucose intolerance.

In contrast, the present study successfully reflected the status of glucose intolerance using three metrics: CP/I, IGI, and PNI. To assess the ability of these indices to respond to β-cell function or insulin clearance, this study evaluated CP/I, IGI, and PNI using ROC curve analysis. This showed that IGI was better than PNI for evaluating β-cell function and that CP/I could reflect insulin clearance. Previous studies have shown that IGI is a determinant of blood glucose levels 2 hours after oral glucose administration [14]; therefore, we recommend using IGI to assess pancreatic β-cell function. CP/I is a good indicator for evaluating the body's ability to clear insulin. Although numerous studies have demonstrated a reduction in IGI among patients with IGR and T2DM, the present study further elucidates the critical role of IGI in evaluating pancreatic β-cell function.

## 5 LIMITATIONS

This study had some limitations. The IGR group was categorized by glucose levels after oral glucose tolerance testing and included patients with IFG and IGT, despite differences in their pathologies. Therefore, further examination of these indicators across different glucose tolerance states is required. In addition, studies comparing c-peptide measurements have shown significant differences between immunoassays [30], which may affect clinical diagnosis and the interpretation of study results. A notable limitation of the present study was the random recruitment of participants, which resulted in imbalances in BMI, gender composition, and age distribution across the groups. This demographic discrepancy could have introduced an unintended confounding factor into the interpretation of the study outcomes. Furthermore, the relatively small sample size in this study may have led to insufficient statistical power and limited the generalizability of the findings, and this demographic discrepancy, combined with the limited sample volume, could have exerted an unintended confounding effect on the interpretation of the study outcomes.

## CONCLUSION

**IGI and CP/I can be used to Evaluate Islet Function**

The results showed that among the different methods of assessing insulin resistance and pancreatic β-cell function, CP/I, IGI, and PNI were more sensitive in reflecting impaired glucose tolerance. Our results demonstrate that IGI and CP/I are good indicators for evaluating the body's β-cell function and insulin clearance, respectively. However, these indicators cannot replace traditional markers such as blood glucose and insulin.

## Figures and Tables

**Tables 1:** Classification of glucose metabolic status (WHO 1999 diagnostic criteria).

Glucose Metabolic Status	Venous Plasma Glucose (mmol/L)	
-	Fasting Glucose (mmol/L)	2 h Glucose (mmol/L)
NGT	<6.1	<7.8
IFG	≥6.1, <7.0	<7.8
IGT	<7.0	≥7.8, <11.1
T2DM	≥7.0	≥11.1

**Table 2:** Basic information on the study population.

Group	NGT	IGR	T2DM	*P*
N	30	43	42	-
Men	4 (13.333%)	12 (27.907%)	16 (38.095%)	-
Women	26 (86.667%)	31 (72.093%)	26 (61.905%)	-
Age	36 ^c^ (30.25~47.50)	39 ^c^ (30.00~54.00)	53.5 ^a, b^ (36.00~65.00)	0.002
BMI (kg/m^2^)	25.834 ^b^ (22.052~27.740)	27.763 ^a, c^ (25.393~31.956)	26.112 ^b^ (23.438~28.273)	0.004
Fasting glucose (mmol/L)	5.100 ^b, c^ (4.720~5.320)	5.860 ^a, b^ (5.170~6.200)	7.650 ^a, b^ (7.383~7.983)	<0.0001
2 h glucose (mmol/L)	6.390 ^b, c^ (5.508~7.133)	8.720 ^a, c^ (7.950~10.140)	17.180 ^a, b^ (14.295~18.575)	<0.0001
Fasting insulin (mU/L)	9.900 (6.250~17.200)	14.550 (9.300~16.600)	14.100 (8.025~16.775)	ns
2 h insulin (mU/L)	75.200 ^b^ (41.550~100.600)	95.000 ^a, c^ (57.500~156.300)	66.900 ^b^ (32.200~98.275)	0.01

**Note:** Values are medians (interquartile range). Compared with normal glucose tolerance (NGT) group, a P<0.05; compared with impaired glucose tolerance regulation (IGR) group, b P<0.05; compared with type 2 diabetes mellitus (T2DM) group, c P<0.05. BMI: body mass index.

**Table 3:** Assessment of insulin resistance and β-cell function by different indicators.

-	Groups			*P*	Post-hoc		
-	NGT	IGR	T2DM		NGT *vs*. IGR	NGT *vs*. T2DM	IGR *vs*. T2DM
HOMA-IR	2.356 (1.473~4.067)	3.539 (2.290~4.550)	4.832 (2.547~5.615)	<0.0001	ns	<0.0001	ns
CP/I	0.0005 (0.0002~0.001)	0.003 (0.001~0.007)	0.007 (0.005~0.009)	<0.0001	0.001	<0.0001	0.022
HOMA-β	143.356 (90.048~230.626)	122.549 (92.174~185.135)	63.710 (39..906~80.692)	<0.0001	ns	<0.0001	<0.0001
PI/I	2.995 (1.930~4.124)	4.331 (2.481~5.630)	9.754 (6.216~14.679)	<0.0001	ns	<0.0001	<0.0001
PI/CP	5.877 (2.038~13.090)	1.269 (0.585~2.781)	1.343 (0.855~2.913)	<0.0001	<0.0001	0.001	ns
IGI	180.780 (94.974~252.796)	77.389 (43.509~145.288)	29.785 (15.4122~52.088)	<0.0001	0.004	<0.0001	<0.0001
CPI	0.026 (0.009~0.177)	0.041 (-0.004~0.107)	0.031 (0.004~0.096)	ns	-	-	-
PNI	209.040 (122.620~270.485)	105.469 (63.702~167.532)	57.545 (10.111~103.181)	<0.0001	0.004	<0.0001	0.038
PI-AUC/I-AUC	2.883 (2.247~3.798)	3.006 (1.961~3.946)	5.313 (3.199~8.539)	<0.0001	ns	<0.0001	<0.0001
PI-AUC/CP-AUC	7.403 (1.816~13.567)	1.866 (1.219~4.183)	1.456 (0.921~3.082)	<0.0001	0.005	<0.0001	ns
I-AUC/G-AUC	70.093 (48.372~84.275)	62.846 (42.201~82.505)	24.458 (13.037~35.659)	<0.0001	ns	<0.0001	<0.0001
CP-AUC/G-AUC	25.471 (14.670~112.301)	66.748 (35.812~149.220)	69.432 (39.985~110.174)	0.005	0.043	ns	ns
PI-AUC/G-AUC	188.130 (132.250~264.154)	161.893 (109.278~223.000)	117.380 (77.879~184.318)	<0.0001	ns	0.002	ns

**Note:** Values are median (interquartile range). **Abbreviation:** HOMA-IR: Homeostatic model assessment for insulin resistance; HOMA-β: Homeostasis model assessment-β; G: glucose; I: Insulin; CP: C-peptide; PI: proinsulin; CP/I: the ratio of C-peptide to insulin; PI/I: The ratio of proinsulin to insulin; PI/CP: The ration of proinsulin to C-peptide; IGI: insulinogenesis index (∆I30/∆G30); CPI: C-peptide index (∆C30/∆G30); PNI: proinsulin index (∆PI30/∆G30).

## Data Availability

The data sets used and/or analysed during this study are available from the corresponding author upon request.

## LIST OF ABBREVIATIONS

OGTT: Oral Glucose Tolerance Tests
NGT: Normal Glucose Tolerance
IGR: Impaired Glucose Regulation
T2DM: Type 2 Diabetes Mellitus
IGI: Insulinogenic Index
CPI: C-Peptide Generation Index
PNI: Proinsulinogenic Index
CP/I: Fasting-State Ratios of C-Peptide to Glucose
PI/I: Fasting-State Ratios of Proinsulin to Insulin
PI/CP: Fasting-State Ratios of Proinsulin to C-Peptide
AUC: Area Under the Curve
IFG: Impaired Fasting Glucose
IGT: Impaired Glucose Tolerance
HOMA-IR: Homeostatic Model Assessment for Insulin Resistance
HOMA-β: Homeostasis Model Assessment-β
ROC Curve: Receiver Operating Characteristic Curve

## STANDARDS OF REPORTING

STROBE guidelines and methodology were followed.
