# Supplementary material for: Comparison of Islet Function in People With Different Glucose Metabolic States
Source: Endocr Metab Immune Disord Drug Targets. 2026 May 21;26:E18715303436762. doi: 10.2174/0118715303436762260508092256 (PMC13595326; doi:10.2174/0118715303436762260508092256)
Supplement: Supplementary file 1 [file EMIDDT-26-E18715303436762_SD1.pdf]

Supplementary Material

Comparison of Islet Function in People With Different Glucose Metabolic States

Guiling Li[1], Yixian Liu[2,#], Xiaoxuan Liu[1], Huijing Luo[1], Huimin Zhou[1], Xu Huang[3], Qian He[1], Jinhong Li[1,#], Yanv Ren[1], Xiaofang Zhang[1] and Zuoliang Dong[1,*]

[1]Department of Clinical Laboratory, Tianjin Medical University General Hospital, No.154 Anshan Road, Heping District, Tianjin 300052, China; [2]Bacteriological Disease Prevention and Disinfection Institute, Hebei Province Center for Disease Prevention and Control, No. 97 Huai'an Donglu, Shijiazhuang 050021, Hebei, China; [3]School of Medical Imaging, Tianjin Medical University, No.1 Guangdong Road, Hexi District, Tianjin, 300204, China

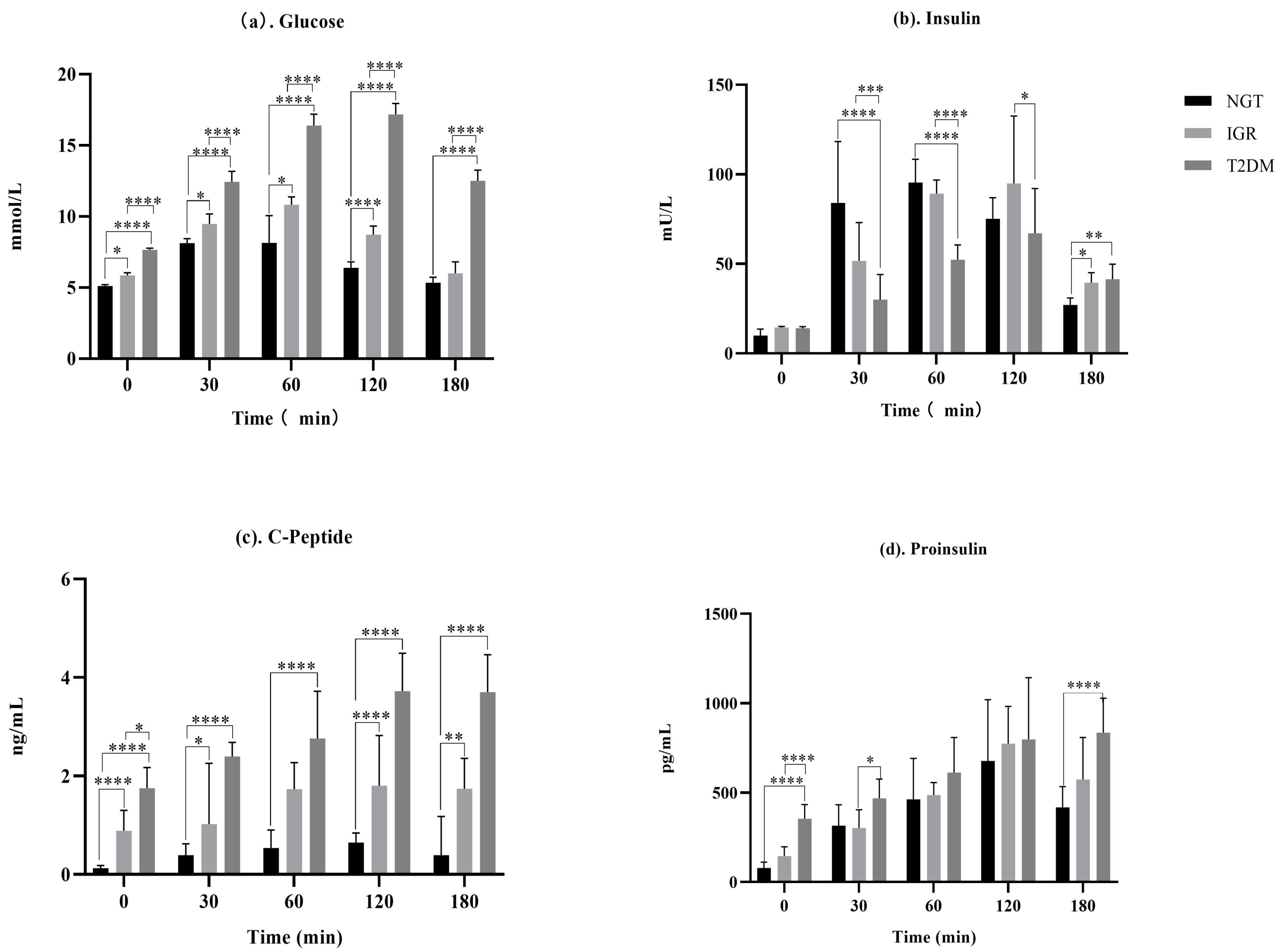

Fig. (S1).

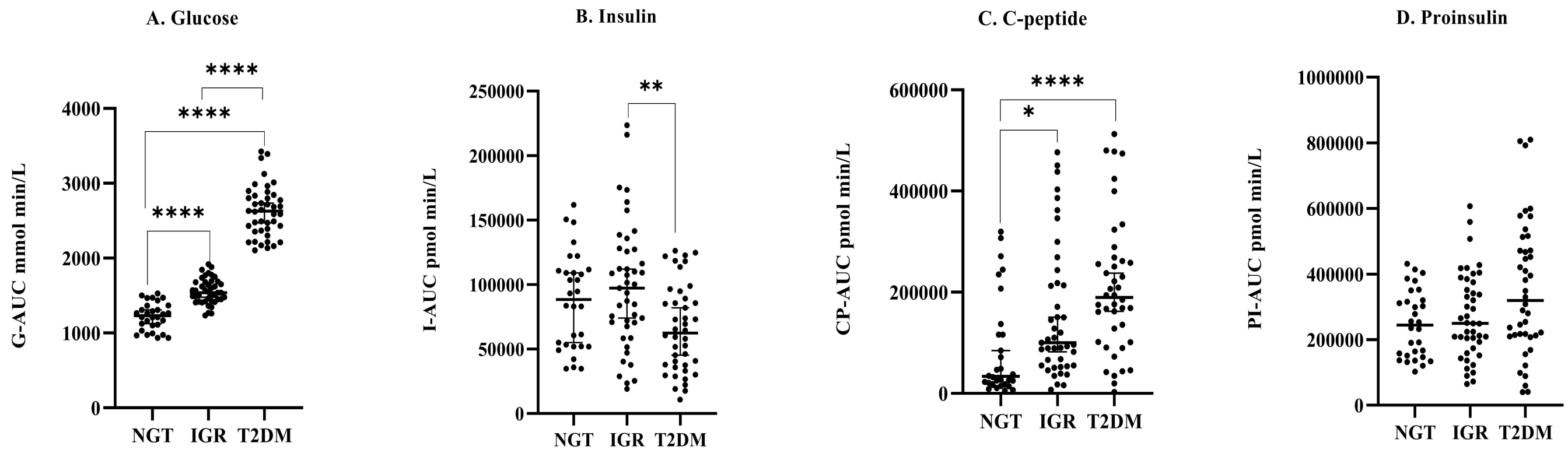
A. Glucose
G-AUC mmol min/L
4000
3000
2000
1000
0
****
****
****
NGT
IGR
T2DM
B. Insulin
I-AUC pmol min/L
250000
200000
150000
100000
50000
0
**
NGT
IGR
T2DM
C. C-peptide
CP-AUC pmol min/L
600000
400000
200000
0
****
*
NGT
IGR
T2DM
D. Proinsulin
PI-AUC pmol min/L
1000000
800000
600000
400000
200000
0
NGT
IGR
T2DM

Fig. (S2).
